# Sequencing immunotherapy in locally advanced head and neck squamous cell carcinoma: a narrative clinical-translational review

**DOI:** 10.3389/fonc.2026.1895540

**Published:** 2026-09-03

**Authors:** Ruey-Long Hong

**Affiliations:** 1 Department of Medical Oncology, National Taiwan University Cancer Center, Taipei, Taiwan; 2 Department of Oncology, National Taiwan University Hospital, Taipei, Taiwan

**Keywords:** chemoradiotherapy, head and neck squamous cell carcinoma, immunotherapy, neoadjuvant therapy, PD-1 blockade, postoperative therapy, treatment sequencing, tumor-draining lymph nodes

## Abstract

**Background:**

Immune checkpoint inhibitors have entered curative-intent treatment of locally advanced head and neck squamous cell carcinoma, but randomized trials have shown divergent results across definitive, perioperative, and postoperative settings.

**Methods:**

We reviewed pivotal randomized phase II–III trials and key translational studies to examine how treatment sequence relative to radiation and surgery, together with anatomic context, influences efficacy.

**Results:**

Concurrent immunotherapy with definitive chemoradiotherapy has repeatedly failed to improve primary endpoints or locoregional control, a pattern consistent with a myeloid-rich “macrophage sink” model in bulky irradiated tumors. By contrast, perioperative therapy reduced distant failure, consistent with a model of immune priming in intact tumor-draining lymph nodes, whereas postoperative therapy improved locoregional control after removal of gross disease in a “clean bed” of microscopic residual disease. Delayed maintenance after definitive therapy did not demonstrate benefit. HPV status, PD-L1 expression, tumor volume, elective nodal irradiation, and primary subsite may further modulate benefit and contribute to subgroup heterogeneity.

**Conclusions:**

In locally advanced head and neck squamous cell carcinoma, immunotherapy benefit appears sequence- and context-dependent. Neoadjuvant treatment may preferentially reduce distant risk, postoperative intensification may reinforce local control in selected high-risk patients, and routine concurrent use with definitive chemoradiotherapy should remain investigational.

## Introduction – the clinical paradox

For nearly three decades, curative management of locally advanced head and neck squamous cell carcinoma (LA-HNSCC) has centered on surgery, radiation, and concurrent chemoradiotherapy (CRT) (1). Despite incremental advances, survival has plateaued, approximately half of patients recur or metastasize, and the addition of docetaxel-based induction chemotherapy has not consistently improved outcomes (2). The advent of immune checkpoint inhibitors (ICIs) (3) raised the prospect that moving immunotherapy “upstream” into curative settings—where host immunity is more intact—might improve outcomes (4).

However, highly anticipated trials have produced an apparent contradiction. Large phase III efforts adding ICIs concurrently to CRT (e.g., KEYNOTE-412, JAVELIN Head and Neck 100) (5, 6) did not meet their primary endpoints, whereas perioperative and postoperative strategies (KEYNOTE-689, NIVOPOSTOP) (7, 8) have reported meaningful benefits. These divergent results may reflect, at least in part, the interaction between treatment sequence and anatomic context. We therefore propose a testable model in which concurrent high-dose radiation to bulky disease may generate a wound-healing, myeloid-rich microenvironment (“macrophage sink”) that limits local checkpoint efficacy, whereas neoadjuvant or properly sequenced approaches may preserve or exploit intact tumor-draining lymph nodes to support systemic antitumor priming. This framework is intended as a hypothesis-generating interpretation of the current evidence rather than a definitive mechanistic conclusion.

Article type and scope. This manuscript is a narrative, hypothesis-generating clinical-translational review that synthesizes randomized phase II–III clinical trials and selected translational studies relevant to immunotherapy sequencing with surgery and radiation in LA-HNSCC over the period from 2019 through February 2026. The aim is to provide a balanced overview of current curative-intent trial results while proposing a testable framework in which treatment sequence, tumor bulk, and tumor-draining lymph node integrity may help explain divergent patterns of benefit across resectable, postoperative high-risk, and unresectable settings. Throughout the review, evidence-supported observations are distinguished from biologically plausible interpretations, and areas of uncertainty are explicitly highlighted.

The key trials informing the proposed sequencing framework are summarized in **Tables 1A, B**. **Table 1A** focuses on unresected definitive, sequential, and maintenance strategies, whereas **Table 1B** summarizes perioperative and postoperative strategies in resectable or high-risk resected disease. Publication status and endpoint hierarchy are included to distinguish mature phase III evidence from exploratory, subgroup, or conference-level data.

**Table 1A:** Definitive, sequential, and maintenance strategies in unresected LA-HNSCC.

Trial	Setting/Population	Immunotherapy Timing and Treatment Backbone	Publication Status/Data Maturity	Primary Endpoint Result	Selected Secondary, Exploratory, *Post Hoc*, or Translational Findings	Interpretation for Sequencing Framework
KEYNOTE-412 Phase III	Unresected LA-HNSCC; mixed HPV/p16 population	Pembrolizumab priming dose → concurrent pembrolizumab + cisplatin/RT → maintenance pembrolizumab vs placebo + cisplatin/RT	Primary report: full peer-reviewed article; long-term follow-up: conference/follow-up report	Primary EFS narrowly missed the prespecified superiority boundary: HR 0.83, 95% CI 0.68–1.03; p=0.0429	Secondary/follow-up: Long-term follow-up favored pembrolizumab for EFS, but benefit appeared driven mainly by reduced distant metastasis; LRC was not clearly improved.	Suggests systemic activity may persist despite limited locoregional benefit during concurrent definitive CRT; supports the hypothesis that bulky irradiated disease may be less permissive for local checkpoint efficacy
JAVELIN Head and Neck 100 Phase III	Unresected LA-HNSCC	Avelumab priming dose → concurrent avelumab + cisplatin/RT → maintenance avelumab vs placebo + cisplatin/RT	Full peer-reviewed article	PFS did not improve; trial terminated early for futility: HR 1.21, 95% CI 0.93–1.57	Secondary/exploratory: OS numerically favored control; exploratory PD-L1 subgroup signals were reported.	Does not support routine concurrent PD-L1 blockade with definitive CRT; raises the hypothesis that antibody class, target biology, and radiation timing may influence outcome
GORTEC-REACH, cisplatin-fit cohort Phase III	Unresected LA-HNSCC; cisplatin-fit patients	Avelumab + cetuximab/RT vs standard cisplatin/RT	Conference abstract/meeting report	PFS inferior with avelumab + cetuximab/RT: HR approximately 1.40	Locoregional progression was increased in the experimental arm	Suggests IO-based substitution for cisplatin is not supported in cisplatin-fit patients receiving definitive RT; reinforces the importance of cytotoxic treatment backbone
GORTEC-REACH, cisplatin-unfit cohort Phase III	Unresected LA-HNSCC; cisplatin-unfit patients	Avelumab + cetuximab/RT vs cetuximab/RT	Conference abstract/meeting report	PFS not significantly improved	No clear LRC improvement; distant failure signal reported in some analyses	Hypothesis-generating; suggests systemic effects may differ from locoregional effects, but interpretation is limited by setting and publication maturity
CompARE, durvalumab arm Phase III	Oropharyngeal cancer; intermediate- and high-risk groups; HPV/p16 risk strata relevant	Durvalumab induction → standard CRT → adjuvant durvalumab vs CRT alone	Conference abstract/meeting report; subgroup findings exploratory/*post hoc*	Overall OS in intention-to-treat population did not improve	Exploratory subgroup data suggested possible benefit in higher-risk/HPV-negative patients and lack of benefit in intermediate-risk HPV-positive patients	Supports risk-stratified interpretation by HPV/p16 status, smoking exposure, and baseline risk; subgroup findings should be considered hypothesis-generating
Zandberg et al. Randomized Phase II	Unresected LA-HNSCC; concurrent vs sequential pembrolizumab strategy	Concurrent pembrolizumab starting before CRT vs sequential pembrolizumab beginning after CRT	Full peer-reviewed article with exploratory translational analyses	1-year PFS favored the sequential arm: 84% vs 71%	Secondary/exploratory: LRC favored sequential therapy: 96% vs 64%. Exploratory/translational: paired biopsies showed increased macrophages and PD-L1-positive macrophages in the concurrent arm.	Supports the hypothesis of sequence-dependent differences between concurrent and sequential immunotherapy; translational findings are exploratory and not definitive proof of mechanism
IMvoke010 Phase III	High-risk LA-HNSCC after definitive local therapy; included surgical and non-surgical patients with no progression before maintenance	Delayed atezolizumab maintenance beginning after completion of definitive therapy vs placebo	Full peer-reviewed article	EFS did not improve: HR 0.94, 95% CI 0.70–1.26	Heterogeneous prior local therapy and delayed treatment initiation complicate interpretation	Suggests delayed maintenance after heterogeneous definitive therapy does not provide clear benefit; does not exclude benefit from earlier postoperative or sequence-optimized strategies

**Table 1B:** Perioperative and postoperative strategies in resectable or high-risk resected LA-HNSCC.

Trial	Setting/Population	Immunotherapy Timing and Treatment Backbone	Publication Status/Data Maturity	Primary Endpoint Result	Selected Secondary, Exploratory, *Post Hoc*, or Translational Findings	Interpretation for Sequencing Framework
KEYNOTE-689 Phase III	Resectable stage III/IVA LA-HNSCC	Neoadjuvant pembrolizumab → surgery → adjuvant pembrolizumab with postoperative RT/CRT as indicated vs surgery → standard adjuvant therapy	Full peer-reviewed article	EFS improved: HR 0.73, 95% CI 0.58–0.92	Secondary/exploratory: Benefit appeared driven mainly by reduced distant failure; LRC was not clearly improved. Pathologic response: pCR was 3% and mPR was 9.3%.	Supports perioperative pembrolizumab in selected resectable patients; pattern is consistent with, but does not prove, systemic immune priming before surgery
NIVOPOSTOP/GORTEC 2018–01 Phase III	High-risk resected LA-HNSCC after macroscopic complete surgery; high-risk pathology	Nivolumab + postoperative cisplatin/RT followed by adjuvant nivolumab vs postoperative cisplatin/RT	Full peer-reviewed article	DFS improved: HR 0.76, 95% CI 0.60–0.98	Secondary/exploratory: LRC improved; no clear DMFS benefit was observed. Biomarker/subgroup: benefit was observed across PD-L1 strata without a clear monotonic CPS relationship.	Supports postoperative intensification in selected high-risk resected patients; pattern is consistent with improved local tumor-bed control after removal of gross disease
CAMORAL Randomized Phase II	Resectable LA-HNSCC; predominantly HPV-negative and oral cavity cancer population	Neoadjuvant camrelizumab + albumin-bound paclitaxel + carboplatin → surgery → adjuvant therapy	Conference presentation/abstract-level data; not yet mature full publication in current manuscript	EFS improved in reported phase II analysis: HR approximately 0.34	Secondary/exploratory: pCR was approximately 25% and mPR approximately 48%. Exploratory: early DMFS and LRC signals were reported.	Encouraging but hypothesis-generating; supports further study of intensified neoadjuvant chemoimmunotherapy and possible organ-preservation strategies in selected phenotypes

CRT, chemoradiotherapy; CPS, combined positive score; DFS, disease-free survival; DMFS, distant metastasis-free survival; EFS, event-free survival; HNSCC, head and neck squamous cell carcinoma; HPV, human papillomavirus; IO, immunotherapy; LA-HNSCC, locally advanced head and neck squamous cell carcinoma; LRC, locoregional control; mPR, major pathological response; OS, overall survival; pCR, pathological complete response; PD-1, programmed death 1; PD-L1, programmed death ligand 1; PFS, progression-free survival; RT, radiotherapy.

Endpoint labels indicate the level at which findings were reported when ascertainable from available publications or conference reports. “Secondary” denotes protocol-specified non-primary endpoints; “exploratory” denotes analyses not used for primary efficacy determination; “*post hoc*” denotes subgroup analyses not prospectively powered for definitive inference; and “translational” denotes biological correlative analyses.

## Methods for literature identification and study selection

This article was conducted as a narrative, hypothesis-generating review rather than a formal systematic review or meta-analysis. PubMed/MEDLINE and major oncology conference proceedings, including ASCO, ESMO, and AACR, were reviewed for randomized immunotherapy trials and major clinical reports published or presented between January 2019 and February 2026. Earlier foundational or contextual studies were included when they informed standard therapy, HPV/p16 risk interpretation, macrophage biology, tumor-draining lymph node biology, or radiotherapy-immunology interactions. Search concepts included “head and neck squamous cell carcinoma,” “locally advanced,” “immunotherapy,” “PD-1,” “PD-L1,” “chemoradiotherapy,” “neoadjuvant,” “perioperative,” “postoperative,” “maintenance,” “elective nodal irradiation,” and “tumor-draining lymph node.”

Randomized phase II–III trials integrating immune checkpoint inhibitors into curative-intent treatment of resectable, postoperative high-risk, or unresectable LA-HNSCC were prioritized. Translational studies were selected if they provided mechanistic insight relevant to treatment sequencing, radiotherapy timing, myeloid remodeling, dendritic cell trafficking, or lymph node-dependent immune priming. Conference abstracts and follow-up reports were included only when they materially informed the current evidence base and are explicitly identified according to publication status.

Data extracted included treatment setting, sequence relative to surgery and radiation, treatment backbone, sample size, primary endpoint, hazard ratios with confidence intervals where available, and selected secondary or exploratory outcomes including locoregional control, distant metastasis-related outcomes, pathological response, and toxicity. Because the review was narrative in intent, no formal risk-of-bias scoring tool was applied; instead, differences in trial design, population, endpoint definitions, and publication maturity were considered qualitatively when interpreting cross-trial comparisons.

## Clinical trial evidence and hypothesis-generating biological interpretation

**Figure 1:**
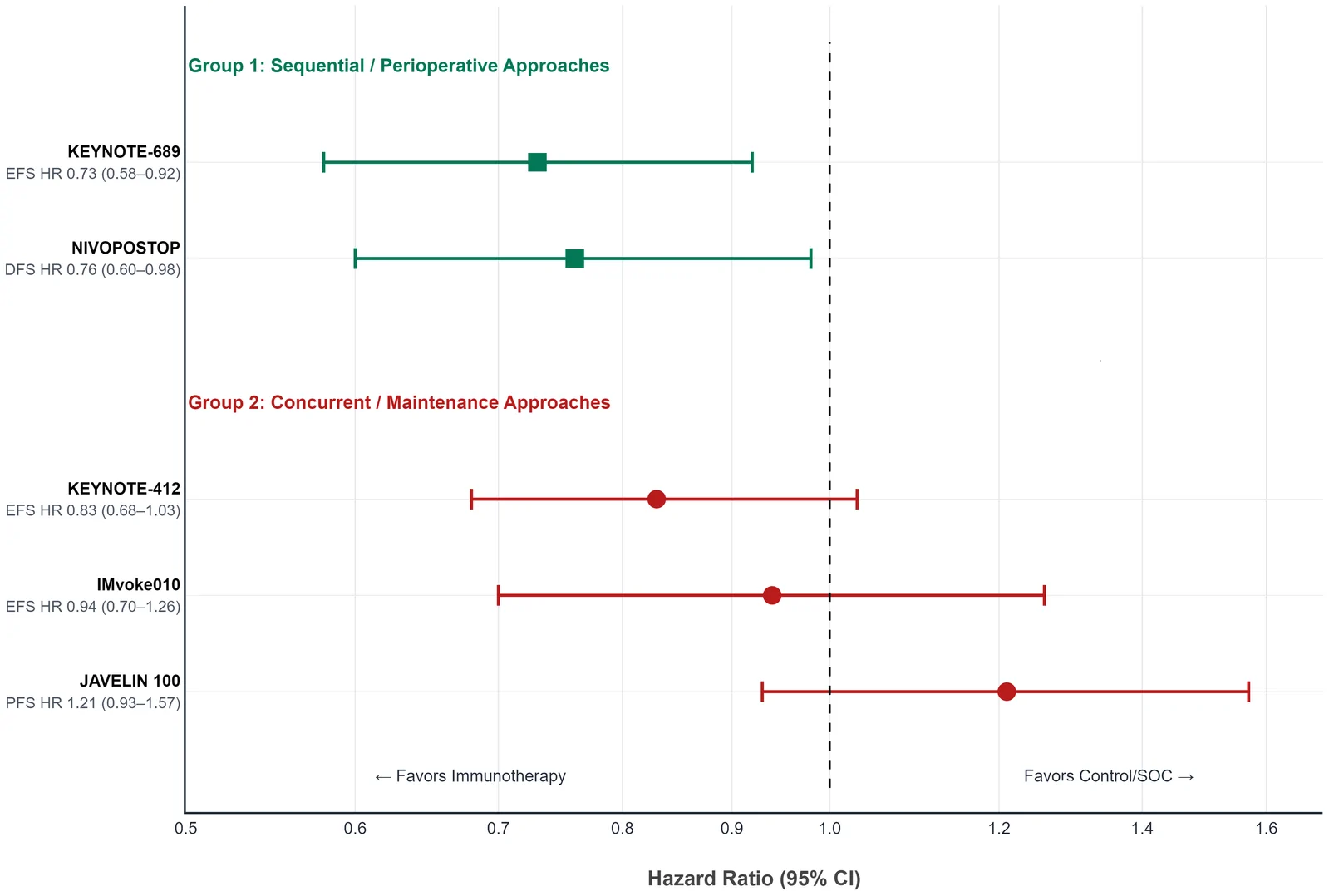
Forest plot synthesis of primary efficacy endpoints across pivotal phase III immunotherapy trials in locally advanced head and neck squamous cell carcinoma. The plot visualizes the clinical divergence between sequential/perioperative administration strategies (squares) and concurrent/maintenance strategies (circles). The central markers represent the point estimate for the hazard ratio (HR), and the horizontal error bars represent the 95% confidence intervals (CI). Trials utilizing perioperative or selected sequential approaches show hazard ratios favoring benefit, whereas trials using concurrent or delayed maintenance strategies have generally not demonstrated comparable advantage. Because these studies differ in setting, patient population, and treatment backbone, this figure is intended as a visual summary of trial-level patterns rather than a formal comparative meta-analysis. CI, confidence interval; DFS, disease-free survival; EFS, event-free survival; HR, hazard ratio; PFS, progression-free survival.

The clinical landscape of immunotherapy in LA-HNSCC is characterized by a recurring contrast: concurrent definitive chemoradiotherapy (CRT) strategies have generally not produced consistent improvements in primary endpoints or locoregional control, whereas perioperative and selected postoperative strategies have shown clinically meaningful benefit in defined settings (**Figure 1**). These trial patterns provide clinical observations that may help generate biological hypotheses regarding how timing, tumor bulk, and nodal immune architecture influence immunotherapy efficacy. However, the following comparisons should be interpreted as hypothesis-generating rather than definitive mechanistic proof.

### Concurrent intensification: negative and mixed trials

The hypothesis that adding PD-1/PD-L1 inhibitors to definitive CRT would synergize with radiation-induced antigen release was tested in major trials including KEYNOTE-412, JAVELIN Head and Neck 100, and GORTEC-REACH (5, 6, 9). These studies differed in design and treatment backbone, but collectively they did not establish a consistent role for routine concurrent immunotherapy with definitive CRT in unselected LA-HNSCC populations.

KEYNOTE-412 (Pembrolizumab + CRT) (5) was a near-positive phase III trial in which the primary event-free survival (EFS) analysis favored pembrolizumab but did not cross the prespecified superiority boundary. Longer follow-up later showed an EFS advantage, but the apparent benefit was driven mainly by reduced distant metastasis rather than by a clear improvement in locoregional control (LRC) (10). This pattern suggests that pembrolizumab may have retained systemic activity while not substantially improving local tumor-bed control during concurrent definitive CRT. One possible explanation is that the irradiated microenvironment of bulky disease may limit local checkpoint efficacy, but this remains an inference rather than a proven mechanism.

JAVELIN Head and Neck 100 (Avelumab + CRT) (6) was terminated early for futility, with the experimental arm showing numerically inferior progression-free survival compared with placebo. Avelumab differs from pembrolizumab in that it is an IgG1 anti-PD-L1 antibody with antibody-dependent cellular cytotoxicity (ADCC) capability (11). In a radiation-inflamed tumor bed, PD-L1 may be expressed not only by tumor cells but also by activated immune and myeloid populations. Therefore, one hypothesis is that concurrent use of an ADCC-competent anti-PD-L1 antibody could have unintended effects on immune effector compartments. This possibility remains speculative and should be interpreted cautiously, but it underscores the need to consider antibody class, target biology, and radiation timing when designing immunoradiotherapy combinations.

Together, KEYNOTE-412 and JAVELIN Head and Neck 100 suggest that concurrent checkpoint blockade during definitive CRT may not reliably augment local control in bulky irradiated disease. These findings are consistent with, but do not prove, the possibility that radiation-associated inflammatory and myeloid programs may partially counteract local checkpoint activity.

The GORTEC-REACH Cisplatin-Fit Cohort (Avelumab + Cetuximab/RT vs CRT) (9) further illustrates the importance of treatment backbone. In cisplatin-fit patients, replacing high-dose cisplatin with avelumab plus cetuximab during radiation resulted in inferior PFS and OS, with a marked increase in locoregional progression. These results suggest that immunotherapy-based substitution for cisplatin is not supported in cisplatin-fit patients receiving definitive radiation. Rather than functioning as a reliable radiosensitizer, immune checkpoint blockade may require an appropriate disease setting, tumor burden, and immune context to produce benefit.

### The adjuvant paradox: “clean bed” versus delayed maintenance

**Figure 2:**
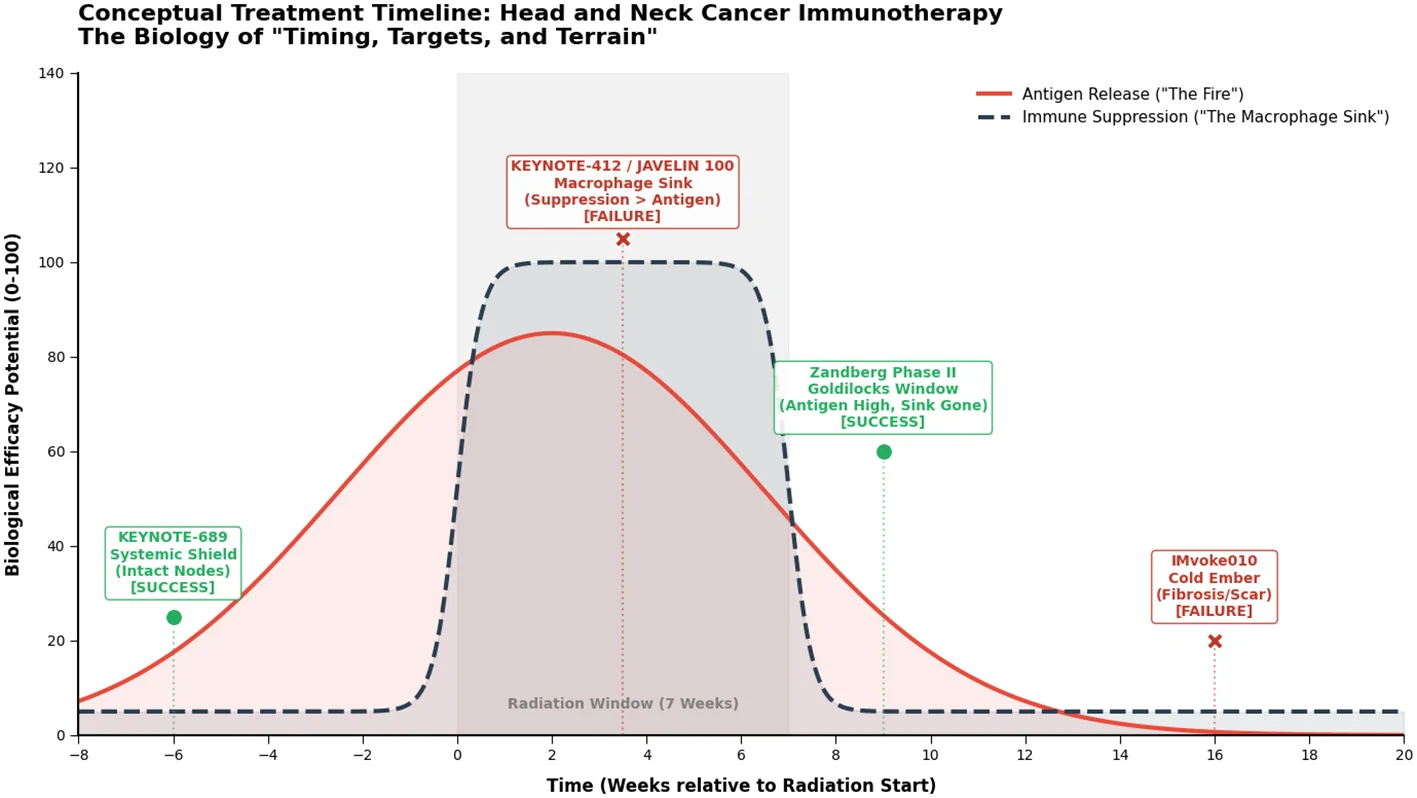
Conceptual timeline of treatment sequence relative to phases of radiation-associated inflammation, wound healing, and fibrosis. The schematic illustrates the relative positioning of neoadjuvant, concurrent, early postoperative/adjuvant, and delayed maintenance approaches discussed in the manuscript, including the approximate timing of IMvoke010 initiation. This figure is intended as a hypothesis-generating representation of biologically distinct treatment windows rather than a validated temporal model.

The postoperative and post-definitive settings illustrate that “adjuvant” immunotherapy is not a single biological context. NIVOPOSTOP (8) and IMvoke010 (12) both evaluated immunotherapy after local therapy, but they differed substantially in timing, disease status, prior treatment, and residual tumor burden (**Figure 2**).

In NIVOPOSTOP (Nivolumab + Postoperative CRT) (8), patients with high-risk resected disease received nivolumab in combination with postoperative cisplatin-based chemoradiotherapy. This approach improved disease-free survival and was associated with improved locoregional control. The pattern is consistent with the possibility that removal of macroscopic tumor burden creates a postoperative setting in which checkpoint blockade can cooperate with radiation and chemotherapy to address microscopic residual disease. However, this interpretation remains inferential; the contribution of surgery, cisplatin, radiation timing, immune context, and wound-healing biology cannot be isolated from this trial alone.

In contrast, IMvoke010 (Atezolizumab Maintenance) (12) evaluated atezolizumab maintenance after completion of definitive therapy and did not demonstrate an EFS benefit. One possible explanation is that delayed initiation after resolution of active antigen release and tissue remodeling may be less favorable for immune reactivation. However, the interpretation of IMvoke010 is also complicated by heterogeneity in prior local therapy, inclusion of both surgical and non-surgical patients, variation in residual risk, and the requirement that patients had no progression before maintenance therapy. Thus, the negative result should not be interpreted as a universal failure of postoperative immunotherapy, but rather as evidence that delayed maintenance after heterogeneous definitive therapy may not be sufficient in this setting.

Taken together, NIVOPOSTOP and IMvoke010 support the hypothesis that timing and residual disease context may influence postoperative immunotherapy efficacy. Early intervention in a microscopic residual-disease setting may differ substantially from delayed maintenance in a clinically disease-free, post-radiation milieu.

### Perioperative strategies and subsite-specific uncertainty

The neoadjuvant and perioperative setting provides a distinct immune context because the primary tumor and tumor-draining lymph nodes remain intact at the time of initial checkpoint exposure. This principle was tested most prominently in KEYNOTE-689 (7) and CAMORAL (13).

KEYNOTE-689 evaluated perioperative pembrolizumab in resectable LA-HNSCC and demonstrated improved EFS (7). The benefit appeared to be driven largely by reduced distant failure, with no clear improvement in locoregional control. This pattern is consistent with a model in which neoadjuvant immune exposure may contribute to systemic antitumor priming before surgery, potentially through intact tumor-draining lymph nodes. However, alternative explanations should also be considered, including postoperative continuation of pembrolizumab, differences in perioperative event ascertainment, baseline risk composition, and timing of detection of early systemic disease. Therefore, the “systemic shield” concept should be viewed as a testable model rather than a proven mechanism.

The hypopharyngeal subgroup finding in KEYNOTE-689 should be interpreted with particular caution. Although subgroup analyses suggested a possible lack of benefit or trend toward harm in hypopharyngeal cancer, the number of patients was small and confidence intervals were wide. This signal is clinically important and should prompt prospective evaluation, but it should not be used alone to define treatment eligibility or exclusion outside the context of clinical judgment and future trial validation.

CAMORAL (Camrelizumab plus chemotherapy followed by surgery and adjuvant therapy) (13) explored an intensified neoadjuvant chemoimmunotherapy strategy in a randomized phase II setting enriched for HPV-negative and oral cavity disease. The reported EFS and pathological response results are encouraging and suggest that deeper neoadjuvant response may be achievable with combination approaches. However, because CAMORAL remains a smaller phase II study and publication maturity should be clearly specified, these findings should be interpreted as hypothesis-generating. The possibility that deep response could support future organ-preservation strategies, particularly in laryngeal or hypopharyngeal cancer, warrants prospective testing with functional endpoints such as laryngectomy-free survival and tracheostomy-free survival.

Overall, the perioperative data support the concept that neoadjuvant or perioperative immunotherapy can produce clinically meaningful benefit in selected resectable LA-HNSCC populations. The dominant pattern of distant failure reduction is compatible with systemic immune priming, but the precise contribution of intact lymphatic architecture, postoperative continuation therapy, and patient selection remains to be defined.

## Unresectable disease: sequencing signals from the Zandberg trial

The randomized phase II trial by Zandberg et al. (14) provides one of the most informative datasets for examining immunotherapy sequence in unresectable LA-HNSCC because it compared concurrent pembrolizumab starting before CRT with sequential pembrolizumab beginning after CRT. Although the study was hypothesis-generating and not powered for definitive between-arm comparisons, the observed differences in clinical and translational outcomes provide important signals regarding the interaction between radiation timing and checkpoint blockade.

The sequential arm demonstrated a 1-year progression-free survival (PFS) rate of 84% compared with 71% in the concurrent arm. With longer follow-up, locoregional control (LRC) also appeared more favorable in the sequential arm (96% vs. 64%; HR 0.11, 95% CI 0.01–0.89), while 4-year PFS numerically favored the sequential strategy (69% vs. 49%; HR 0.55, 95% CI 0.25–1.22). These findings should be interpreted cautiously because of sample size and trial design, but they support further prospective evaluation of sequential or delayed immunotherapy approaches in unresectable disease.

Exploratory paired tumor biopsies obtained before treatment and during week 2 of CRT showed increased macrophages, PD-L1-positive macrophages, and PD-L1-positive tumor cells in the concurrent arm but not in the sequential arm. These translational observations are consistent with the possibility that early checkpoint blockade given during active radiation-induced tissue injury may be associated with a myeloid-enriched tumor microenvironment. This finding supports, but does not prove, the proposed “macrophage sink” model.

Several non-mutually exclusive mechanisms may contribute to reduced local efficacy of concurrent immunoradiotherapy. First, radiation-induced inflammatory signals may promote recruitment or polarization of tumor-associated macrophages and other myeloid populations. Second, if these recruited cells express PD-1 or PD-L1, they may alter the effective distribution or functional availability of PD-1/PD-L1 blockade within the tumor microenvironment. Third, myeloid-rich wound-healing programs may suppress cytotoxic T-cell activity and contribute to radioresistance. These mechanisms remain biologically plausible and are supported by selected translational data (15–17), but they require prospective validation in human tissue studies.

The Zandberg findings also provide a useful lens through which to reinterpret larger definitive CRT trials. In KEYNOTE-412, long-term follow-up suggested an EFS benefit that appeared driven mainly by reduced distant metastasis rather than improved locoregional control (10). This pattern is consistent with the possibility that pembrolizumab retained systemic activity while failing to meaningfully enhance local radiation effects in bulky irradiated disease. In JAVELIN Head and Neck 100 (6), the lack of benefit and numerical trend toward worse outcomes may reflect differences in checkpoint target, antibody isotype, treatment timing, patient selection, or radiation-associated immune modulation. However, these trial-level observations cannot establish a specific mechanism.

**Figure 3:**
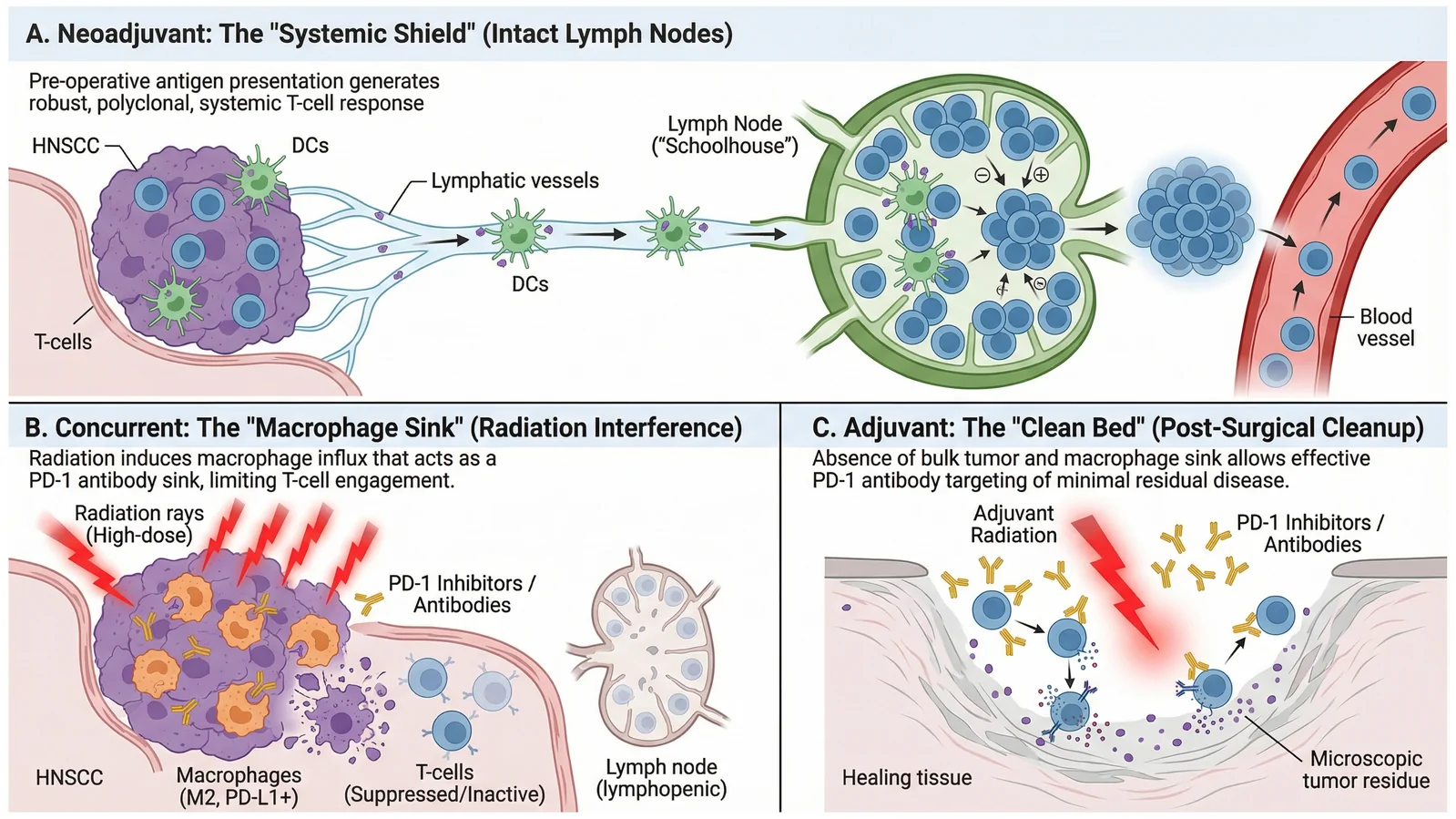
Hypothesis-generating conceptual model of treatment sequence and anatomic context in locally advanced head and neck squamous cell carcinoma. **(A)** Neoadjuvant treatment with intact tumor-draining lymph nodes may permit CCR7-positive dendritic-cell trafficking, nodal antigen presentation, and systemic T-cell priming, represented by the proposed ‘systemic shield’ model. **(B)** Concurrent checkpoint blockade with irradiation of bulky disease may be associated with radiation-induced myeloid recruitment and a macrophage-enriched tumor microenvironment, represented by the proposed ‘macrophage sink’ model. **(C)** After surgical removal of gross disease, postoperative immunotherapy with chemoradiotherapy may act in a microscopic residual-disease setting, represented by the proposed ‘clean bed’ model. Elements of this schematic are derived from trial observations and selected translational studies and should be interpreted as biologically plausible, hypothesis-generating models rather than established mechanisms.

Taken together, the available unresectable-disease data support a hypothesis that concurrent checkpoint blockade during definitive CRT may be less favorable than sequential approaches in some patients, particularly when bulky tumor and elective nodal irradiation are present. One possible explanation is that local myeloid enrichment within the irradiated tumor bed and nodal irradiation–associated lymphocyte depletion may jointly limit effective antitumor immunity (**Figure 3**). This remains a testable model rather than a settled conclusion, and future trials should directly evaluate optimized sequencing strategies with embedded translational endpoints.

## Resectable and postoperative disease: distinct patterns of benefit

The positive trials in resectable and postoperative LA-HNSCC—KEYNOTE-689 and NIVOPOSTOP—provide an opportunity to examine how treatment setting may influence the pattern of immunotherapy benefit. Both trials met their primary endpoints, but the distribution of benefit differed: KEYNOTE-689 appeared to reduce distant failure more than locoregional failure, whereas NIVOPOSTOP appeared to improve locoregional control without a clear distant metastasis benefit. These differences are consistent with a sequence- and context-dependent model, although they should not be interpreted as proving distinct mechanisms because the trials differed in design, eligibility, risk composition, treatment delivery, and event ascertainment.

### KEYNOTE-689: perioperative immunotherapy and distant failure

KEYNOTE-689 demonstrated improved event-free survival with perioperative pembrolizumab in resectable LA-HNSCC. The benefit appeared to be driven primarily by reduced distant metastasis, while locoregional control was not clearly improved. This pattern is consistent with the proposed model that neoadjuvant immune exposure, delivered while the primary tumor and tumor-draining lymph nodes remain intact, may support systemic antitumor priming. Translational data from Saddawi-Konefka et al. (18) suggest that dendritic-cell trafficking from tumor to sentinel lymph nodes, including CCR7+ dendritic-cell migration, may be relevant to response to sequenced immunoradiotherapy (18). However, the extent to which this mechanism explains the clinical benefit observed in KEYNOTE-689 remains uncertain.

Several alternative or complementary explanations should be considered. The observed distant metastasis benefit may reflect not only preoperative priming but also postoperative continuation of pembrolizumab, suppression or delayed emergence of early systemic disease, differences in recurrence ascertainment, or baseline risk heterogeneity. Therefore, the “systemic shield” concept should be interpreted as a biologically plausible and testable model rather than as a proven explanation of the KEYNOTE-689 results.

### NIVOPOSTOP: postoperative immunotherapy and locoregional control

NIVOPOSTOP evaluated the addition of nivolumab to postoperative cisplatin-based CRT in high-risk resected LA-HNSCC and demonstrated improved disease-free survival (8). In contrast to KEYNOTE-689, the benefit appeared to be driven primarily by improved locoregional control, with no clear reduction in distant metastasis. This pattern is consistent with the possibility that removal of gross tumor burden creates a postoperative setting in which immunotherapy can cooperate with CRT to address microscopic residual disease.

This interpretation should also be viewed cautiously. The improved locoregional control may reflect the combined effects of surgical debulking, postoperative radiation, cisplatin, nivolumab, wound-healing biology, and patient selection. Likewise, the absence of a clear distant metastasis benefit may be related to nodal dissection, elective nodal irradiation, baseline risk, or insufficient capacity for new systemic immune priming after surgery and nodal treatment. Preclinical and translational studies suggest that tumor-draining lymph nodes and elective nodal irradiation can influence systemic antitumor immunity (15, 17), but the direct contribution of these factors to the NIVOPOSTOP clinical results remains to be established.

### Working interpretation: sequence and context may shape patterns of benefit

Taken together, KEYNOTE-689 and NIVOPOSTOP support a hypothesis-generating clinical principle: the setting in which immunotherapy is delivered may influence whether benefit is expressed predominantly as reduced distant failure or improved locoregional control. In resectable disease, neoadjuvant or perioperative treatment may be most relevant when systemic micrometastatic risk is a major concern and tumor-draining lymph nodes remain intact. In postoperative high-risk disease, immunotherapy may be most relevant for reinforcement of locoregional control when gross tumor burden has been removed and the therapeutic target is microscopic residual disease. These interpretations remain inferential and require prospective validation with translational endpoints capable of distinguishing immune priming, tumor-bed effects, and nodal immune contributions.

## Modifying factors: patient selection, HPV/p16 status, and PD-L1

Although treatment sequence and disease setting are central to the framework proposed in this review, patient and tumor characteristics also influence interpretation of clinical trial results. HPV/p16 status, PD-L1 expression, smoking history, tumor volume, nodal burden, anatomical subsite, and treatment backbone may all modify apparent benefit from immunotherapy. These factors should therefore be considered as contextual variables rather than binary determinants of response.

### HPV/p16 status and trial population composition

HPV-associated oropharyngeal cancer is often more responsive to standard CRT than HPV-negative disease (19), but HPV-positive disease is not uniformly low risk. Outcomes vary according to smoking exposure, T category, N category, tumor volume, comorbidity, and treatment context. Therefore, the inclusion of HPV-positive or otherwise favorable-risk patients in unselected intensification trials may reduce the apparent absolute benefit of additional systemic therapy, but this should not be interpreted as evidence that HPV-positive patients cannot benefit from immunotherapy-based strategies.

In KEYNOTE-412, approximately one quarter of patients were p16-positive, which may have influenced the overall effect size because many favorable-risk patients can achieve durable control with standard CRT. Conversely, exploratory subgroup findings from CompARE (**Table 1A**) (20) suggested that higher-risk HPV-negative patients may have derived greater benefit from immunotherapy-containing sequencing, whereas intermediate-risk HPV-positive patients did not clearly benefit. These observations are hypothesis-generating and should be interpreted cautiously because subgroup analyses are susceptible to imbalance, limited power, and differences in baseline risk. They nevertheless support the need for future trials to stratify by HPV/p16 status, smoking exposure, tumor burden, and risk group rather than relying on broad all-comer enrollment.

### PD-L1 as an enrichment marker rather than a binary rule

PD-L1 combined positive score (CPS) may enrich for benefit from immune checkpoint blockade in some HNSCC settings, but its predictive value appears context-dependent. CPS is influenced by assay platform, tissue sampling site, tumor heterogeneity, immune-cell composition, timing of biopsy, and prior treatment exposure. Its relevance may also differ between neoadjuvant treatment of gross disease, definitive chemoradiotherapy, and postoperative treatment of microscopic residual disease (21).

In KEYNOTE-689, the small PD-L1 CPS <1 subgroup had limited pathological response, suggesting that baseline immune activation may be relevant to neoadjuvant activity. However, the subgroup was very small, and this observation should not be overinterpreted as a definitive exclusion criterion. In KEYNOTE-412, longer-term benefit appeared more evident in the CPS >1 population, which is consistent with PD-L1 enrichment but does not establish CPS as an absolute requirement for benefit in definitive therapy.

The postoperative setting may be different. In NIVOPOSTOP, benefit was observed across PD-L1 strata without a clear monotonic relationship between CPS and outcome. This suggests that after surgical removal of gross tumor burden, PD-L1 may be less reliable as a single selection tool, possibly because the therapeutic target is microscopic residual disease within a different immune and wound-healing context. Accordingly, PD-L1 should be viewed as one component of risk and response assessment rather than as a universal binary biomarker.

Overall, HPV/p16 status and PD-L1 CPS should be incorporated into trial design and interpretation, but neither should be treated as a standalone determinant of immunotherapy benefit. Future studies should prospectively evaluate composite selection strategies that integrate viral status, smoking exposure, tumor volume, nodal burden, residual disease risk, PD-L1 expression, circulating tumor DNA, and early pathological response.

## Toxicity and practical considerations

The integration of immune checkpoint inhibitors (ICIs) into curative-intent LA-HNSCC treatment introduces immune-related adverse events and logistical challenges that may affect surgery, radiation delivery, chemotherapy tolerance, and multidisciplinary coordination. Because curative-intent therapy is highly time-sensitive, toxicity should be considered not only by frequency and severity but also by its potential to disrupt planned local treatment.

In KEYNOTE-689, perioperative pembrolizumab increased potentially immune-mediated adverse events compared with standard therapy. Although most patients proceeded to surgery, treatment-related adverse events were associated with a higher frequency of surgical delay in the pembrolizumab arm. These findings suggest that neoadjuvant immunotherapy is feasible in selected resectable patients, but requires careful perioperative monitoring for endocrinopathies, hepatitis, pneumonitis, and other immune-related toxicities that could jeopardize the timing of definitive surgery.

In NIVOPOSTOP, the addition of nivolumab to postoperative cisplatin-based CRT improved disease-free survival but was associated with a high overall burden of grade 3–4 treatment-related adverse events and an increase in serious adverse events. Signals for renal and thyroid toxicity are particularly relevant when PD-1 blockade is combined with cisplatin and radiation. These data support close monitoring of renal function, hydration status, endocrine parameters, nutritional status, and treatment adherence when immunotherapy is layered onto an already intensive postoperative CRT backbone.

The toxicity profile of concurrent immunotherapy with definitive CRT also warrants caution. In the randomized phase II Zandberg trial, dose-limiting toxicities occurred in the concurrent arm but not in the sequential arm, including severe airway-related toxicity. Although the sample size was small, this observation is clinically relevant in head and neck cancer, where radiation-associated mucosal edema, dysphagia, aspiration risk, and airway compromise can have immediate consequences. It raises the possibility that concurrent immune activation during peak mucosal inflammation may amplify local toxicity in selected patients.

Overall, available toxicity data support careful attention to treatment timing, patient selection, and multidisciplinary logistics when integrating ICIs into curative-intent LA-HNSCC therapy. While separating immunotherapy from the period of maximal radiation-associated inflammation may be biologically and clinically attractive, this remains a hypothesis that requires prospective evaluation. Future trials should prospectively collect timing-specific toxicity, surgical delay, feeding-tube dependence, airway intervention, renal toxicity, endocrine toxicity, and treatment-completion endpoints alongside efficacy outcomes.

## Cross-disease perspective on sequencing

Although this review focuses on LA-HNSCC, similar sequencing patterns have been observed in other tumor types. In thoracic oncology, trials that incorporated immunotherapy concurrently with CRT did not consistently improve outcomes (22, 23), whereas sequential consolidation after CRT established standards of care in stage III non–small cell lung cancer (24, 25) and limited-stage small cell lung cancer (26). In gastroesophageal cancer, adjuvant nivolumab after resection improved disease-free survival, paralleling the concept that removal of gross tumor burden may create a more permissive environment for postoperative immunotherapy (27).

These parallels should be interpreted cautiously, because HNSCC differs from thoracic and gastroesophageal malignancies in several important respects, including the extent of elective nodal irradiation, patterns of lymphatic drainage, and the frequent need to balance local control with organ preservation. Nevertheless, the broader oncology experience supports the view that treatment sequence relative to radiation and surgery may be a clinically relevant modifier of immunotherapy benefit and toxicity.

## Limitations of cross-trial inference

The sequence-dependent framework proposed in this review is derived primarily from indirect comparisons across randomized trials conducted in biologically and clinically distinct settings. Although these comparisons are useful for generating hypotheses, they should not be interpreted as proving that treatment sequence is the sole or dominant causal determinant of benefit. KEYNOTE-412, JAVELIN Head and Neck 100, GORTEC-REACH, Zandberg et al., NIVOPOSTOP, IMvoke010, KEYNOTE-689, CAMORAL, and CompARE differed substantially in patient population, resectable versus unresectable setting, HPV/p16 distribution, pathological risk composition, treatment backbone, checkpoint target, antibody isotype, inclusion of surgery, timing and duration of immunotherapy exposure, radiation fields, endpoint definitions, and follow-up maturity. These differences limit the certainty with which any single mechanistic explanation can be assigned across studies.

Several examples illustrate this complexity. The contrast between KEYNOTE-689 and NIVOPOSTOP is consistent with a model in which neoadjuvant treatment preferentially supports systemic immune priming, whereas postoperative treatment preferentially improves local tumor-bed control after removal of gross disease. However, these trials also differed in baseline risk, pathological selection, cisplatin use, treatment adherence, perioperative timing, and recurrence ascertainment, any of which may have contributed to the observed divergence in distant versus locoregional benefit. Similarly, the negative or unfavorable results of concurrent definitive trials may reflect more than treatment sequence alone, including differences in tumor burden, treatment backbone, elective nodal irradiation, HPV composition, PD-L1 distribution, and checkpoint biology.

Publication maturity also deserves consideration. Some evidence discussed in this review derives from conference-level reports, follow-up presentations, or exploratory analyses rather than final peer-reviewed full-length publications. These data are clinically informative but inherently less secure than mature primary reports and should be interpreted accordingly. For these reasons, the present synthesis should be viewed as a hypothesis-generating clinical-translational model intended to organize current evidence and guide future prospective testing, rather than as a definitive comparative assessment of sequence across trials.

## Clinical impact and future directions

**Figure 4:**
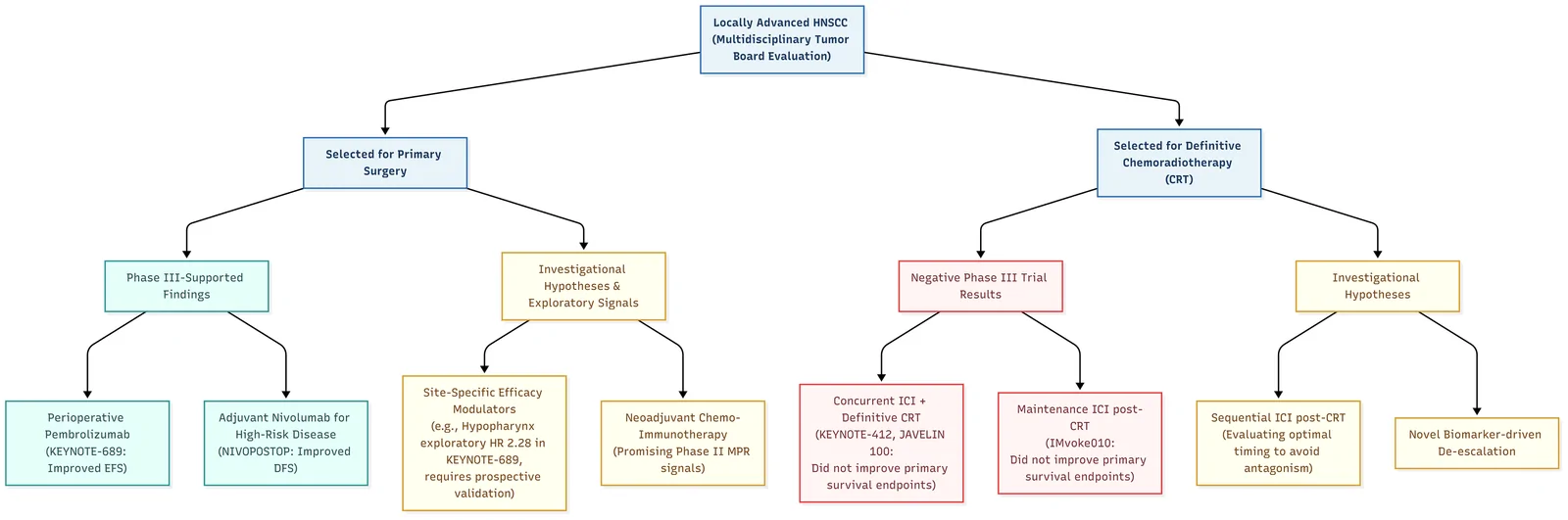
Non-prescriptive evidence framework for immunotherapy sequencing in locally advanced head and neck squamous cell carcinoma.This figure summarizes current randomized evidence and hypothesis-generating biological interpretations without providing treatment eligibility rules or site-specific exclusions. Phase III-supported findings are shown separately from negative or non-supportive randomized trial results and from investigational hypotheses requiring prospective validation. KEYNOTE-689 supports perioperative pembrolizumab in patients selected for a surgery-based management strategy, but should not be interpreted as applying automatically to all resectable disease. NIVOPOSTOP supports postoperative nivolumab with cisplatin-based chemoradiotherapy in selected high-risk resected patients. Concurrent immunotherapy with definitive chemoradiotherapy and delayed maintenance immunotherapy after definitive therapy have not demonstrated routine benefit in the specific regimens and settings tested. Exploratory hypopharyngeal subgroup findings are insufficient to define treatment exclusion or establish hyperprogression risk.

The divergent results of curative-intent immunotherapy trials in locally advanced head and neck squamous cell carcinoma (LA-HNSCC) suggest that immune checkpoint blockade should not be viewed as a uniformly additive intervention across all treatment settings. Rather, available evidence supports a more nuanced interpretation in which the timing of immunotherapy relative to surgery and radiotherapy, the presence or absence of gross tumor burden, and the integrity of tumor-draining lymph nodes may influence the pattern of benefit. A non-prescriptive evidence framework for immunotherapy sequencing is shown in **Figure 4**. The figure is intended to summarize current randomized trial observations, negative trial results, and investigational hypotheses rather than to provide a treatment algorithm. In particular, exploratory subgroup findings, including those in hypopharyngeal cancer, should not be used alone to define treatment eligibility or exclusion.

In resectable disease, perioperative PD-1 blockade is supported by phase III evidence and appears to preferentially reduce distant failure. This observation is consistent with the possibility that neoadjuvant immune exposure, delivered while tumor antigen and lymphatic architecture are still present, may support systemic antitumor priming. However, the relative contributions of preoperative priming, postoperative continuation of therapy, event ascertainment, and baseline risk composition remain incompletely defined. Subsite-specific signals, including the exploratory hypopharyngeal subgroup findings, should therefore be interpreted cautiously and require prospective validation.

In high-risk resected disease, postoperative immunotherapy combined with CRT has also shown phase III benefit, with improvement driven primarily by locoregional control. This pattern is consistent with a model in which surgical removal of gross disease creates a postoperative setting more permissive for immune-mediated sterilization of microscopic residual tumor. Nevertheless, this interpretation remains inferential, and the relative impact of tumor debulking, radiation timing, cisplatin exposure, nodal treatment, and postoperative wound-healing biology requires further study.

For unresectable disease treated with definitive chemoradiotherapy, randomized trials have not consistently supported routine concurrent addition of immune checkpoint inhibitors. These negative or mixed results are compatible with the hypothesis that bulky irradiated tumors and elective nodal irradiation may limit effective antitumor immunity, but they do not establish a single mechanism of resistance. In this setting, sequence-optimized strategies—such as delayed, sequential, or sandwich approaches—remain investigational and should be tested prospectively with embedded translational endpoints.

Several clinical and biological modifiers should be incorporated into future trial design. HPV/p16 status should be treated as a risk-stratifying variable rather than a binary favorable-risk label, because outcomes vary by smoking exposure, T/N category, tumor volume, and treatment context. PD-L1 CPS may enrich for benefit in selected settings but should not be interpreted as a universal exclusion criterion, particularly in postoperative high-risk disease. Tumor volume, nodal architecture, elective nodal irradiation, primary subsite, and timing of recurrence assessment should also be prospectively captured to clarify whether they modify immunotherapy efficacy.

Future studies should explicitly test the timing-and-terrain framework proposed here. Priorities include randomized evaluation of optimized sequencing strategies in unresectable disease; incorporation of translational endpoints such as myeloid dynamics, dendritic-cell trafficking, T-cell receptor clonality, and circulating tumor DNA; refinement of elective nodal irradiation timing and field design where oncologically safe; and prospective validation of organ-preservation strategies in laryngeal and hypopharyngeal cancers using functional endpoints such as laryngectomy-free survival and tracheostomy-free survival. Biomarker-informed de-escalation or escalation strategies should also be explored, particularly when pathological response or molecular residual disease assessment can identify patients at substantially different risks of recurrence.

In summary, immunotherapy benefit in LA-HNSCC appears to be shaped by more than drug selection alone. Current evidence supports a testable model in which treatment sequence, tumor burden, nodal immune architecture, and clinical setting may influence whether checkpoint blockade primarily affects distant failure, locoregional control, or neither.

Prospective studies designed around these variables will be required to determine which elements of this framework are causal, predictive, or merely associative.
